# Supplementary material for: Heparin-based hydrogel scaffolding alters the transcriptomic profile and increases the chemoresistance of MDA-MB-231 triple-negative breast cancer cells
Source: Biomater Sci. 2020 Feb 13;8(10):2786–96. doi: 10.1039/c9bm01481k (PMC7497406; doi:10.1039/c9bm01481k)
Supplement: Supplementary file 2 [file BM-008-C9BM01481K-s002.zip › Supplementary File 4/HP-BhydrogelvControl/Pathways/my_analysis.Gsea.1545201079907/HALLMARK_SPERMATOGENESIS.html]

Details for gene set HALLMARK\_SPERMATOGENESIS[GSEA]

|  || Dataset | expr.class.cls#HYD\_versus\_CONTROL.class.cls#HYD\_versus\_CONTROL\_repos |
| Phenotype | class.cls#HYD\_versus\_CONTROL\_repos |
| Upregulated in class | CONTROL |
| GeneSet | HALLMARK\_SPERMATOGENESIS |
| Enrichment Score (ES) | -0.24657264 |
| Normalized Enrichment Score (NES) | -1.0307014 |
| Nominal p-value | 0.36679536 |
| FDR q-value | 0.40975723 |
| FWER p-Value | 0.992 |
Table: GSEA Results Summary

  

Fig 1: Enrichment plot: HALLMARK\_SPERMATOGENESIS      
 Profile of the Running ES Score & Positions of GeneSet Members on the Rank Ordered List

  

| PROBE | DESCRIPTION (from dataset) | GENE SYMBOL | GENE\_TITLE | RANK IN GENE LIST | RANK METRIC SCORE | RUNNING ES | CORE ENRICHMENT || 1 | CNIH2 | na |  |  | 39 | 3.711 | 0.0673 | No |
| 2 | CHFR | na |  |  | 430 | 2.434 | 0.0923 | No |
| 3 | GFI1 | na |  |  | 554 | 2.275 | 0.1284 | No |
| 4 | MAST2 | na |  |  | 760 | 2.067 | 0.1562 | No |
| 5 | PGS1 | na |  |  | 775 | 2.053 | 0.1939 | No |
| 6 | MTOR | na |  |  | 1611 | 1.525 | 0.1787 | No |
| 7 | CLPB | na |  |  | 1778 | 1.455 | 0.1972 | No |
| 8 | PHKG2 | na |  |  | 2109 | 1.310 | 0.2044 | No |
| 9 | NF2 | na |  |  | 2587 | 1.147 | 0.2008 | No |
| 10 | ACRV1 | na |  |  | 3125 | 1.004 | 0.1915 | No |
| 11 | DDX25 | na |  |  | 3553 | 0.907 | 0.1861 | No |
| 12 | SLC12A2 | na |  |  | 4547 | 0.702 | 0.1473 | No |
| 13 | SCG5 | na |  |  | 5507 | 0.551 | 0.1074 | No |
| 14 | LPIN1 | na |  |  | 5826 | 0.510 | 0.1003 | No |
| 15 | GAD1 | na |  |  | 5904 | 0.499 | 0.1056 | No |
| 16 | PCSK4 | na |  |  | 6041 | 0.482 | 0.1075 | No |
| 17 | EZH2 | na |  |  | 6297 | 0.445 | 0.1025 | No |
| 18 | HSPA2 | na |  |  | 6430 | 0.426 | 0.1036 | No |
| 19 | HSPA1L | na |  |  | 6971 | 0.364 | 0.0821 | No |
| 20 | NEFH | na |  |  | 6997 | 0.360 | 0.0875 | No |
| 21 | JAM3 | na |  |  | 8155 | 0.239 | 0.0315 | No |
| 22 | OAZ3 | na |  |  | 8655 | 0.185 | 0.0089 | No |
| 23 | MAP7 | na |  |  | 8695 | 0.182 | 0.0102 | No |
| 24 | PCSK1N | na |  |  | 8793 | 0.167 | 0.0083 | No |
| 25 | MLLT10 | na |  |  | 8821 | 0.164 | 0.0099 | No |
| 26 | GPR182 | na |  |  | 9146 | 0.135 | -0.0045 | No |
| 27 | BRAF | na |  |  | 9238 | 0.124 | -0.0069 | No |
| 28 | CAMK4 | na |  |  | 9346 | 0.111 | -0.0105 | No |
| 29 | MLF1 | na |  |  | 9642 | 0.081 | -0.0244 | No |
| 30 | PAPOLB | na |  |  | 10037 | 0.036 | -0.0443 | No |
| 31 | IP6K1 | na |  |  | 10136 | 0.023 | -0.0490 | No |
| 32 | CCNA1 | na |  |  | 10576 | -0.014 | -0.0717 | No |
| 33 | COIL | na |  |  | 10578 | -0.014 | -0.0715 | No |
| 34 | ZC3H14 | na |  |  | 10830 | -0.043 | -0.0838 | No |
| 35 | TCP11 | na |  |  | 10858 | -0.047 | -0.0843 | No |
| 36 | NCAPH | na |  |  | 10955 | -0.060 | -0.0882 | No |
| 37 | SIRT1 | na |  |  | 10988 | -0.064 | -0.0887 | No |
| 38 | AGFG1 | na |  |  | 11119 | -0.080 | -0.0940 | No |
| 39 | PIAS2 | na |  |  | 11453 | -0.108 | -0.1094 | No |
| 40 | CSNK2A2 | na |  |  | 11527 | -0.116 | -0.1111 | No |
| 41 | CDKN3 | na |  |  | 11708 | -0.139 | -0.1179 | No |
| 42 | NPHP1 | na |  |  | 11843 | -0.157 | -0.1220 | No |
| 43 | CCT6B | na |  |  | 11884 | -0.161 | -0.1211 | No |
| 44 | KIF2C | na |  |  | 11924 | -0.166 | -0.1200 | No |
| 45 | BUB1 | na |  |  | 12059 | -0.180 | -0.1236 | No |
| 46 | PSMG1 | na |  |  | 12254 | -0.207 | -0.1299 | No |
| 47 | TLE4 | na |  |  | 13047 | -0.290 | -0.1659 | No |
| 48 | TOPBP1 | na |  |  | 13157 | -0.307 | -0.1659 | No |
| 49 | SLC2A5 | na |  |  | 13246 | -0.316 | -0.1646 | No |
| 50 | GMCL1 | na |  |  | 13256 | -0.317 | -0.1591 | No |
| 51 | SEPT4 | na |  |  | 13294 | -0.324 | -0.1550 | No |
| 52 | PRKAR2A | na |  |  | 13336 | -0.327 | -0.1510 | No |
| 53 | PARP2 | na |  |  | 14076 | -0.428 | -0.1817 | No |
| 54 | RFC4 | na |  |  | 14127 | -0.430 | -0.1763 | No |
| 55 | VDAC3 | na |  |  | 14267 | -0.455 | -0.1750 | No |
| 56 | DBF4 | na |  |  | 14298 | -0.463 | -0.1680 | No |
| 57 | PHF7 | na |  |  | 14310 | -0.465 | -0.1598 | No |
| 58 | HSPA4L | na |  |  | 14452 | -0.483 | -0.1582 | No |
| 59 | AURKA | na |  |  | 14577 | -0.502 | -0.1553 | No |
| 60 | STRBP | na |  |  | 14731 | -0.528 | -0.1534 | No |
| 61 | SNAP91 | na |  |  | 15228 | -0.601 | -0.1682 | No |
| 62 | RAD17 | na |  |  | 15292 | -0.615 | -0.1600 | No |
| 63 | CDK1 | na |  |  | 16949 | -0.970 | -0.2285 | Yes |
| 64 | CCNB2 | na |  |  | 17048 | -0.999 | -0.2149 | Yes |
| 65 | IFT88 | na |  |  | 17101 | -1.014 | -0.1987 | Yes |
| 66 | ARL4A | na |  |  | 17123 | -1.023 | -0.1807 | Yes |
| 67 | TALDO1 | na |  |  | 17146 | -1.029 | -0.1626 | Yes |
| 68 | GSTM3 | na |  |  | 17268 | -1.074 | -0.1489 | Yes |
| 69 | TSN | na |  |  | 17914 | -1.338 | -0.1577 | Yes |
| 70 | NEK2 | na |  |  | 17957 | -1.352 | -0.1346 | Yes |
| 71 | IDE | na |  |  | 18010 | -1.389 | -0.1114 | Yes |
| 72 | RPL39L | na |  |  | 18082 | -1.430 | -0.0884 | Yes |
| 73 | PEBP1 | na |  |  | 18389 | -1.634 | -0.0739 | Yes |
| 74 | TTK | na |  |  | 18742 | -1.945 | -0.0560 | Yes |
| 75 | CLGN | na |  |  | 18853 | -2.094 | -0.0226 | Yes |
| 76 | STAM2 | na |  |  | 18906 | -2.175 | 0.0153 | Yes |
Table: GSEA details [plain text format]

  

Fig 2: HALLMARK\_SPERMATOGENESIS      
 Blue-Pink O' Gram in the Space of the Analyzed GeneSet

  

Fig 3: HALLMARK\_SPERMATOGENESIS: Random ES distribution      
 Gene set null distribution of ES for **HALLMARK\_SPERMATOGENESIS**
